# Farmers’ market use is associated with fruit and vegetable consumption in diverse southern rural communities

**DOI:** 10.1186/1475-2891-13-1

**Published:** 2014-01-09

**Authors:** Stephanie B Jilcott Pitts, Alison Gustafson, Qiang Wu, Mariel Leah Mayo, Rachel K Ward, Jared T McGuirt, Ann P Rafferty, Mandee F Lancaster, Kelly R Evenson, Thomas C Keyserling, Alice S Ammerman

**Affiliations:** 1 Department of Public Health, Brody School of Medicine, East Carolina University, 600 Moye Blvd., Mailstop 660, Lakeside Annex Modular Unit 8, Room 126, Greenville, NC 27834, USA; 2 Department of Nutrition and Food Science, University of Kentucky, Lexington, KY 40506, USA; 3 Department of Biostatistics, 2435D Health Sciences Building, East Carolina University, Greenville, NC 27834, USA; 4 At the time the manuscript was written: Department of Public Health, East Carolina University, Brody School of Medicine, 1709 West Sixth St, Greenville, NC 27834, USA; 5 Department of Community Health, East Tennessee State University, Johnson City, TN, USA; 6 UNC Center for Health Promotion and Disease Prevention, CB# 7426, 1700 MLK Jr. Blvd, Chapel Hill, NC 27599-7426, USA; 7 Department of Public Health, Brody School of Medicine, East Carolina University, 600 Moye Blvd., Mailstop 660, Lakeside Annex Modular Unit 8, Greenville, NC 27834, USA; 8 Center for Survey Research, East Carolina University, Greenville, NC 27834, USA; 9 Director, Center for Survey Research, Office of Innovation and Economic Development, East Carolina University, Greenville, NC 27834, USA; 10 Department of Epidemiology, 137 East Franklin Street Suite 306, Chapel Hill, NC 27514, USA; 11 Department of Medicine, School of Medicine, University of North Carolina at Chapel Hill, Chapel Hill, NC, USA; 12 Department of Nutrition, Gillings School of Global Public Health, Director, Center for Health Promotion and Disease Prevention, University of North Carolina at Chapel Hill, CB# 7426, Chapel Hill, NC 27599-7426, USA

**Keywords:** Farmers’ market, Fruit and vegetable consumption, Rural communities, Obesity, Random digit dial

## Abstract

**Background:**

While farmers’ markets are a potential strategy to increase access to fruits and vegetables in rural areas, more information is needed regarding use of farmers’ markets among rural residents. Thus, this study’s purpose was to examine (1) socio-demographic characteristics of participants; (2) barriers and facilitators to farmers’ market shopping in southern rural communities; and (3) associations between farmers’ market use with fruit and vegetable consumption and body mass index (BMI).

**Methods:**

Cross-sectional surveys were conducted with a *purposive sample* of farmers’ market customers and a *representative sample* of primary household food shoppers in eastern North Carolina (NC) and the Appalachian region of Kentucky (KY). Customers were interviewed using an intercept survey instrument at farmers’ markets. Representative samples of primary food shoppers were identified via random digit dial (RDD) cellular phone and landline methods in counties that had at least one farmers’ market. All questionnaires assessed socio-demographic characteristics, food shopping patterns, barriers to and facilitators of farmers’ market shopping, fruit and vegetable consumption and self-reported height and weight. The main outcome measures were fruit and vegetable consumption and BMI. Descriptive statistics were used to examine socio-demographic characteristics, food shopping patterns, and barriers and facilitators to farmers’ market shopping. Linear regression analyses were used to examine associations between farmers’ market use with fruit and vegetable consumption and BMI, controlling for age, race, education, and gender.

**Results:**

Among farmers’ market customers, 44% and 55% (NC and KY customers, respectively) reported shopping at a farmers’ market at least weekly, compared to 16% and 18% of NC and KY RDD respondents. Frequently reported barriers to farmers’ market shopping were market days and hours, “only come when I need something”, extreme weather, and market location. Among the KY farmers’ market customers and NC and KY RDD respondents, fruit and vegetable consumption was positively associated with use of farmers’ markets. There were no associations between use of farmers’ markets and BMI.

**Conclusions:**

Fruit and vegetable consumption was associated with farmers’ market shopping. Thus, farmers’ markets may be a viable method to increase population-level produce consumption.

## Background

In the United States, obesity is a major public health problem, disproportionately affecting rural residents [1,2]. Disparate obesity rates among rural residents may be partially due to less access to healthy and fresh foods [3,4]. Policies and environmental changes to increase availability of healthy foods are suggested as contributing solutions to the obesity epidemic [5,6]. In particular, increasing use of farmers’ markets is one potential strategy to increase access to and consumption of fruits and vegetables, which would decrease risk of chronic disease [7]. Thus, farmers’ markets are thought to potentially improve population health and reduce population health disparities; yet little is known about their impact on produce consumption [8].

Farmers’ markets may be a particularly effective strategy to improve access to healthy foods in rural areas, where improving the health status of rural residents may involve more effectively leveraging of the strong rural historical connection to agriculture and farming [9]. However, despite this potential access to sources of fresh produce, research indicates that fruit and vegetable consumption among rural dwellers is lower than among urban dwellers [10,11]. The existing agricultural assets in rural areas may support the establishment of farmers’ markets, which could improve access to healthful foods [9]. There are a number of potential challenges to accomplishing this, which may include low customer base, due to low population density, and limited days and hours of business.

Because of the promise that farmers’ markets hold for improving access to healthy foods in underserved areas, several federal initiatives focus on establishment of and enhancements to farmers’ markets. Such farmers’ market enhancements include increasing access to electronic benefit transfer (EBT) for Supplemental Nutrition Assistance Program (SNAP) participants so that the benefits can be used at local markets, adding cooking demonstrations, taste tests, recipe cards, and increasing public transportation to markets. Evaluation efforts are needed to examine effectiveness of such initiatives on increasing fruit and vegetable consumption [8].

Previous studies have documented the characteristics of people who use farmers’ markets, often finding that farmers’ market customers tend to be of higher socioeconomic status and more likely to be female when compared to the general population [12-14]. However, less is known about specific socio-demographic characteristics of farmers’ market customers in southern rural communities, and how to encourage lower-income residents to use farmers’ markets. In prior work in eastern North Carolina, we found that few individuals receiving federal assistance benefits used farmers’ markets: 17% of SNAP participants and 26% of women receiving federally-funded family planning clinic services reported shopping at farmers’ markets [15,16]. Also, little is known about the barriers and facilitators to farmers’ market shopping [8].

Thus, in order to address the health needs of rural populations by leveraging existing agricultural resources, more information is needed about potential customers and their interaction with farmers’ markets. Therefore, the aims of this study were to (1) describe socio-demographic characteristics of participants; (2) examine barriers and facilitators to farmers’ market shopping in rural communities; and (3) examine associations between farmers’ market access, awareness, and use with the outcomes of fruit and vegetable consumption and body mass index (BMI). To accomplish this, we examined these aims among a *purposive sample* of farmers’ market customers and among a *representative sample* of primary household food shoppers in two geographically and racially diverse rural areas: eastern North Carolina (NC) and the Appalachian region of Kentucky (KY). The findings of this study can guide future farmers’ market initiatives in rural regions, where residents increasingly face nutrition-related health disparities [1,2].

## Methods

### Study setting

This study was conducted in rural eastern NC and Appalachian KY in August 2012 – January 2013. The study areas were chosen as rural areas of the southern United States with high obesity prevalence and low access to fruits and vegetables [1,2,10,11]. While eastern NC and eastern KY were different in terms of residents’ racial composition, obesity and poverty rates were similar in the two regions (Table 1). All portions of this study were reviewed and approved by the Institutional Review Boards of both East Carolina University and University of Kentucky at Lexington.

**Table 1:** Description of study areas in North Carolina and Kentucky

**Characteristics**	**Pitt County, eastern North Carolina**	**Fayette County, eastern Kentucky**	**Jackson County, eastern Kentucky**	**Boone County, eastern Kentucky**
Population estimate (2011 ) [17-20]	171,134	301,569	13,443	121,737
Percent rural dwellers (2010) [21]	25.4	3.1	100	13.3
Percent black (2011) [17-20]	34.4	14.8	0.2	2.9
Percent white (2011) [17-20]	61.4	79.1	98.9	93
Percent living below poverty (2007–2011) [17-20]	24	17.9	35.6	7.2
Percent consuming five or more fruits and vegetables daily(2009*) [22,23]	16.8	22.1	16.4	17.7
Age-adjusted estimates of% Obese Adults (2009) [24]; BMI > = 30 kg/m^2^	35.6	30.7	33.4	30.8
Population density (number of persons per square mile, 2010) [17-20]	257.9	1,042.8	39.1	482.3

*The Pitt County Data are from 2009 BRFSS data [22], and the data for the Kentucky Counties is from “County Group” 2005–2009 BRFSS data [23].

The counties in which this study was set are described in detail in Table 1. Pitt County in eastern NC (2011 population estimate = 171,134) [17] includes a small urban center as its county seat (2011 population estimate = 86,017), and is home to a large regional medical center and a large public University. Three counties of interest in eastern KY included Fayette, Jackson, and Boone Counties. Fayette County was included as it is semi-urban, and somewhat comparable to Pitt County, NC. Jackson and Boone counties were selected as they are both lower-income and semi-rural and provide for geographic diversity in the Appalachian region.

### Random digit dial participant recruitment and survey administration

During September and October 2012, trained interviewers conducted a telephone survey of Pitt County residents. The survey was overseen by the East Carolina University Center for Survey Research, and used a random digit dial (RDD) procedure. Both land lines (n = 887) and cellular telephone lines (n = 500) were included in the purchased sample provided by Survey Sampling International (http://www.surveysampling.com/), and numbers were called during a variety of days and times. Eligibility criteria for participation included being over 18 years of age, a Pitt County resident, and one of the primary food shoppers in the household. The adult who answered the phone and met the eligibility criteria was interviewed. Up to 10 attempts were made to each number in the sample. In addition, up to five scheduled callbacks were made to those we reached at an inconvenient time or did not answer the phone, and one conversion was attempted for each soft refusal. The recruitment script stated “We are … conducting the survey as part of a research study to learn more about where you shop for food, your eating habits, and health”. The script did not state that we were specifically studying farmers’ markets. Of 733 calls made, there were 109 surveys completed, 285 refusals, and 339 not eligible due to language barriers, numbers not in service, not residents of Pitt County, business numbers or no adult being home. The final response rate was 28%. The completed interview usually lasted between 10 and 15 minutes. A $10 gift card incentive was mailed to the participant’s home address upon survey completion. Potential participants were told about the incentive in the second sentence of the recruitment script.

In January, 2013, in Jackson, Boone, and Fayette Counties, Kentucky, trained interviewers conducted a telephone survey with a very similar protocol and questions, overseen by the Survey Research Center at the University of Kentucky at Lexington. Land lines only (no cellular telephone lines) were included in the purchase sample provided by Marketing Systems Group (http://www.m-s-g.com/Web/index.aspx). The RDD procedure ensured that every residential telephone line in these KY counties had an equal probability of being called. Households were further screened to talk to one adult who was a primary food shopper. Up to 15 attempts were made to each number in the sample. In addition, up to ten scheduled callbacks were made to those we reached at an inconvenient time, and one refusal conversion was attempted. Of 425 calls made, there were 149 surveys completed, 236 refusals, and 40 not eligible due to language barriers, or no adult being home, for a final response rate of 39%. The completed interview lasted between 15 and 17 minutes. No gift card incentive was used.

### Farmers’ market customer recruitment and survey administration

In fall 2012, farmers’ market customer intercept surveys were conducted among 70 customers in three markets in Pitt County and among 100 customers in one market per county in Jackson, Boone, and Fayette Counties. Researchers approached potential participants in the markets, asking about their interest in participating. If interested, the survey instrument was interviewer-administered if one customer was interested in participation, or self-administered if more than one customer was interested in participation. A $10 gift card incentive was provided upon NC market survey instrument completion, and no incentives were provided for KY market survey instrument completion. Due to the logistical challenges of conducting surveys in public places and the varying degrees of customer traffic, we did not gather information on the number of persons invited to participate and those who agreed or did not agree to participate. Thus, no response rate is reported.

### Survey instruments

The RDD and customer intercept survey instruments were similar by design. Items from previous survey instruments were used regarding food shopping patterns, Behavioral Risk Factor Surveillance System (BRFSS) survey items, and items specific to farmers’ markets. Survey instruments are available from the first author upon request. Both survey instruments included a series of socio-demographic questions, including age (in years), marital status, race, and education level. Questions assessed participation in the Special Supplemental Nutrition Program for Women, Infants, and Children (WIC), WIC Farmers’ Market Nutrition Program (FMNP), SNAP, and the Senior FMNP. Items also asked about food shopping practices (at discount supercenters, grocery stores, and farmers’ markets), farmers’ market awareness, access, use, barriers, and facilitators.

### Farmers’ market awareness, perceived access, and use

In Pitt County, farmers’ market awareness was measured by providing a list of all known farmer’s markets in the county, and asking if the participant had heard of each market, knew the location (yes/no), and whether the participant shopped at the market. An awareness score was calculated by summing the number of positive responses to each item related to whether the participant had heard of the market (yes/no) and knew the location for each of 14 listed farmers’ markets (yes/no) (maximum score of 28).

Farmers’ market perceived access was measured by asking: “How far from your home is the farmer’s market or produce stand where the primary shopper in your household does most of the shopping? (in minutes and miles)”. This question was not pilot-tested prior to inclusion on the survey.

Farmers’ market use was assessed using one question from the 2013 NC BRFSS, “How often in the past 12 months did you buy fruits or vegetables locally grown such as from a farmer’s market, CSA, roadside stand, or pick-your-own produce farm?” Responses to this question were the main outcome measure in regression analyses and were dichotomized into those visiting markets up to once per month versus 2–3 times or more per month.

Among those completing the farmers’ market intercept survey, we asked how much money was typically spent on produce during a normal shopping trip. We also asked the main reasons he/she shopped at a farmers’ market, reasons that prevent more frequent farmers’ market shopping, and the main reasons preventing purchase of more produce during the current shopping trip. We also asked whether they ate more fruits and vegetables as a result of shopping at the market.

Among RDD respondents only, we asked the likelihood of shopping at farmers’ markets given five scenarios related to enhancements to markets including more public transportation to markets, more nutrition education at markets, more promotion of the market, additional parking, and more vendors. We also asked about potential barriers to fruit and vegetable consumption, including cost, preparation time, and lack of availability.

### Fruit and vegetable consumption and body mass index

In both KY and in NC, we assessed fruit and vegetable consumption among all respondents using a validated Block fruit, vegetable, and fiber screener, provided by NutritionQuest (Berkeley, California) [25,26]. Fruit and vegetable scores were calculated using the standard protocol, summing responses to the 7 fruit and vegetable items. Fruit and vegetable servings per day were calculated using the equation provided by NutritionQuest, using the MyPyramid gender-specific definition of servings per day. BMI was calculated from self-reported height (in pounds) and weight (feet and inches), which is considered a valid method to assess weight status [27,28].

### Statistical analysis

We calculated means and frequencies for descriptive statistics. We qualitatively examined differences between regions and respondent types. We examined associations between the independent variable of farmers’ market use and dependent variables of (1) fruit/vegetable consumption and (2) BMI, in two separate linear regression models. All analyses were adjusted for age, gender, race, and education level and were stratified by region (KY and NC) and by type of survey (farmers’ market customer intercept survey versus RDD telephone survey). Race was not included in KY farmers’ market customer analyses as nearly all customers (99%) were white. Final models for the two dependent variables (fruit and vegetable consumption and BMI) were based upon maximizing the number of observations included in the models, and maximizing R^2^. In the adjusted model with fruit and vegetable consumption as the dependent variable, farmers’ market use was examined as the independent variable. In models with BMI as the dependent variable, farmers’ market use was examined as the independent variable of interest, along with fruit and vegetable consumption. Because few respondents in each of the four groups reported their perceived miles to reach the closest farmers’ market, this variable was not included in any of the regression models.

The Pitt County, NC RDD telephone survey data were weighted by the inverse of the probability of selection at the phone number level, and a post-stratification weighting factor that was developed to sequentially adjusted for landline/wireless coverage in NC, and at the county-level household income, and the race and education of the heads of households. The same weighting method was used for the KY RDD analyses, except that no adjustments were made for landline/wireless coverage (since no cellular telephones were sampled) or for race (since there were very few non-white respondents). A pooled regression analysis of the NC and KY RDD data were conducted to assess the differences between the two states. For this purpose, the weights were further adjusted according to the population sizes of the two states. All analyses were conducted using Statistical Analysis Software (version 9.2, SAS Institute Inc, Cary, North Carolina). Data from the RDD telephone surveys were analyzed using survey-specific procedures in SAS.

## Results

### Farmers’ market customer intercept interview and random digit dial (RDD) survey participant characteristics

The mean ages of participants ranged from 44 to 59 years across the four samples (Table 2). The mean length of time participants had lived at their current residence was 10 years (the minimum of the four samples). The mean BMI among the four samples ranged from 27 – 29 kg/m^2^. Fruit and vegetable servings per day ranged from 3.7 to 7.3 servings, with higher consumption among RDD respondents in both regions, compared to farmers’ market customers in both regions. WIC participation ranged from 3 – 4%, and SNAP participation was 17% and 10%, respectively, in the NC RDD and KY RDD respondents, and there were a higher percentage of SNAP and WIC participants in the RDD sample when compared to participants sampled from the NC and KY farmers’ markets.

**Table 2:** Participant characteristics from farmers’ market intercept interview participants and random digit dial survey participants in Pitt County, eastern North Carolina and in Boone, Jackson, and Fayette Counties, eastern Kentucky

**Characteristic**	**NC farmers’ market intercept interview participants (n = 70)**		**Kentucky farmers’ market intercept interview participants (n = 102)**		**Random digit dial participants in North Carolina (n = 109)**		**Random digit dial participants in Kentucky (n = 149)**	
	**Mean**	**Standard deviation**	**Mean**	**Standard deviation**	**Weighted mean**	**Standard error of the mean**	**Weighted mean**	**Standard error of the mean**
**Age in years**	52.9	18.3	50.8	16.4	43.9	2.2	58.5	2.1
**Length of time at current residence in years**	8.9	9.6	13.9	13.5	10.6	1.8	NA	NA
**Fruit and vegetable Servings per day**	4.3	2.0	3.7	1.8	7.2	0.4	7.3	0.2
**BMI (kg/m**^**2**^**)**	27.9	6.9	28.1	6.1	29.3	1.0	27.4	0.5
	**n**	**%**	**n**	**%**	**n**	**Weighted %, SE of %**	**n**	**Weighted %, SE of %**
**Female, n (%)**	47	67.1	74	72.6	82	68.5, 6.9	111	79.4, 4.2
**Race**								
African American/Other	19	27.5	1	1.0	58	38.5, 6.3	7	5.9, 3.1
White	50	72.5	100	99.0	50	61.5, 6.3	142	94.1, 3.1
**Education**								
College graduate	44	62.9	56	55.5	34	35.4, 6.7	60	35.2, 5.0
Non-college graduate	4	37.1	45	44.6	74	64.6, 6.7	86	64.8, 5.0
**Participation in Federal Food Assistance Programs**								
Special Supplemental Nutrition Program for Women, Infants, and Children (WIC)	2	2.9	1	1.0	10	11.0, 4.5	5	6.8, 3.9
Special Supplemental Nutrition Program for Women, Infants, and Children (WIC) Farmers’ Market Nutrition Program (FMNP)	1	1.4	4	7.8	6	4.9, 2.9	9	7.3, 3.9
Supplemental Nutrition Assistance Program (SNAP)	0	0	5	5.0	25	17.0, 4.7	18	9.5, 4.0
Senior Farmers’ Market Nutrition Program (SFMNP)	1	1.4	4	4.0	6	4.4, 2.5	3	5.8, 3.8

^1^For continuous weighted variables, the cells include the weighted mean and standard error of the mean, For categorical variables, the cells contain the true n, weighted %, standard error of %.

^1^NA = Not asked and thus not available.

We examined differences between the NC and KY RDD respondents, and found, as expected, that significantly more KY respondents were white, when compared to NC respondents. KY respondents were also 14.6 years older and 18 pounds lighter, on average, than NC respondents. There were no other significant differences between the two samples.

### Shopping practices among farmers’ market customer intercept interview and random digit dial (RDD) survey participants

Table 3 shows participant shopping practices among farmers’ market customers and RDD survey participants in eastern NC and in eastern KY. In general, 75 – 88% of respondents stated they shopped at supercenters, and 96 - 97% of respondents reported shopping at supermarkets. Farmers’ market customers tended to shop more frequently at supercenters and supermarkets. Among farmers’ market customers, 36% reported shopping at a farmers’ market once/week in the past 12 months, compared to 12 and 15% of KY and NC RDD respondents, respectively. Participants reported living 12–15 minutes and 7 – 12 miles from the closest farmers’ market. Respondents reported spending between $17.80 and $24.80 per trip on produce at the farmers’ market. The score derived to represent awareness of farmers’ markets in Pitt County ranged from 0 to 28, with farmers’ market customers mean awareness score of 10.7, indicating higher awareness of farmers’ markets, and RDD respondents mean awareness score of 4.4 (out of 28), indicating lower awareness.

**Table 3:** **Participant shopping practices among farmers’ market customers and random digit dial survey participants in Pitt County, eastern North Carolina and in Boone, Jackson, and Fayette Counties, Kentucky**^**1**^

**Shopping practices**	**Farmers’ market customers in NC (n = 70)**		**Farmers’ market customers in Kentucky (n = 102)**		**Random digit dial participants in NC (n = 109)**		**Random digit dial participants in Kentucky (n = 149)**	
	**n**	**%**	**n**	**%**	**n**	**Weighted %, SE of %**	**n**	**Weighted %, SE of %**
**Grocery shopping at supercenter**	61	87.1	76	75.3	94	86.3%, 5.0	123	87.9, 3.0
**Frequency of supercenter shopping**								
a few times per year	8	13.1	21	26.3	9	4.2, 1.4	18	13.6, 4.1
once a month	16	26.2	15	18.8	24	25.3, 6.4	32	29.9, 6.2
2-3 times per month	12	19.7	14	17.5	29	27.7, 6.6	37	26.2. 5.5
one time per week	17	27.9	22	27.5	18	25.5, 6.9	21	13.0, 4.1
2 or more times per week	8	13.1	8	10.0	14	17.2, 5.8	14	14.0, 4.8
**Grocery shopping at supermarket**	68	97.1	97	96.0	107	97.3, 2.3	135	96.1, 1.6
**Frequency of supermarket shopping**								
a few times per year	0	0	6	6.2	1	0.5, 0.5	8	1.6, 0.7
once a month	6	8.8	11	11.3	11	4.8, 1.4	21	13.3, 4.5
2-3 times per month	9	13.2	10	10.3	29	26.5, 6.1	31	25.6, 5.7
one time per week	22	32.4	42	43.3	33	32.1, 6.7	36	22.2, 4.5
2 or more times per week	31	45.6	28	28.9	33	36.1, 7.0	39	37.3, 6.0
**How often in the past 12 months have you purchased fruits and vegetables from a farmers’ market, CSA, etc.?**								
never	4	5.7	1	1.0	56	50.8, 7.0	33	22.4, 5.1
a few times per year	9	12.9	10	10.0	22	16.1, 4.7	58	34.8, 5.5
once a month	7	10.0	16	16.0	8	3.4, 1.2	8	7.0, 3.2
2-3 times per month	19	27.1	18	18.0	9	13.9, 5.5	22	14.5, 3.9
one time per week	25	35.7	36	36.0	13	15.4, 5.4	17	12.1, 3.7
2 or more times per week	6	8.6	19	19.0	1	0.4, 0.4	9	5.8, 3.1
**Barriers to use of farmers’ markets**								
No EBT	1	1.5	1	1.1	1	0.5, 0.5	0	0
Transportation barriers	2	3.0	1	1.1	9	7.1, 3.6	1	3.8, 3.7
Prices	1	1.5	7	7.6	8	7.9, 3.8	8	5.8, 2.4
Extreme weather	4	6.0	19	20.7	NA	NA	5	4.4, 2.2
Parking	1	1.5	1	1.1	NA	NA	1	1.1, 1.1
Market days and hours	14	20.9	13	14.1	16	13.0, 5.0	36	28.6, 6.1
Out of the way	11	16.4	11	12.0	50	52.1, 7.1	21	17.1, 4.8
I only come when I need something	12	17.9	39	42.4	21	19.5, 5.6	13	10.4, 4.3
Other	19	28.4	NA		NA		25	23.7, 5.8
	**Mean**	**Standard deviation**	**Mean**	**Standard deviation**	**Weighted mean**	**Standard error of the mean**	**Weighted mean**	**Standard error of the mean**
**Awareness of farmers’ markets in the county**	10.7	5.9	NA	NA	4.4	0.8	NA	NA
**Perceived distance to closest market (minutes)**	11.7	8.1	14.6	9.6	14.5	1.3	14.3	3.2
**Perceived distance to closest market (miles)**	7.4	6.1	12.6	15.9	8.1	1.6	11.7	3.4
**Dollars spent at farmers’ market per visit**	17.79	10.63	20.64	13.27	24.79	4.0	23.89	2.25

^1^For continuous weighted variables, the cells include the weighted mean (standard error of the mean). For categorical variables, the cells contain the true n (weighted percent, standard error of percent).

Among NC farmers’ market customers, the most frequently reported barriers to shopping at farmers’ markets more were the market days and hours, and “I only come when I need something.” Among KY farmers’ market customers, the most frequently reported barriers were “I only come when I need something” and extreme weather. Among NC RDD respondents, the main things that prevent shopping at farmers’ markets more were market being out of the way (location), and “I only come when I need something”. Among KY RDD respondents, the main barriers were market days and hours and the market being out of the way (location).

We asked the likelihood of Pitt County RDD respondents shopping at farmers’ markets given five scenarios and awareness of farmers’ market promotions, as well as barriers to fruit and vegetable consumption. Approximately 22% of respondents indicated awareness of county-wide efforts to enhance or promote farmers’ markets (data not shown). Table 4 shows that the top scenarios to encourage more farmers’ market shopping were (1) more vendors at the market and (2) more promotion of the market. The top three reasons individuals reported that they do not eat fruits and vegetables were (1) because they often spoil before consumption (53% “agree” or “strongly agree”), (2) the restaurants they frequent do not serve fruits (36% “agree” or “strongly agree”), and (3) they cost too much (35% “agree” or “strongly agree”) (Table 4).

**Table 4:** Pitt County RDD respondents’ likelihood of shopping at farmers’ markets given five scenarios and barriers to fruit and vegetable consumption

**Scenario: “How likely would you be to shop at farmers’ markets if…”**	**Frequency of “more likely”**	**Weighted %, SE of %**	**Frequency of “much more likely”**	**Weighted %, SE of %**
there were public transportation to the market	20	13.9, 4.6	19	15.0, 5.0
more nutrition education activities were at the market	45	42.6, 7.0	13	8.6, 3.6
there were more promotion of the market	46	46.6, 7.0	19	19.2, 5.9
there were more parking at the market	33	23.4, 5.4	11	10.6, 4.8
there were more vendors at the market	49	50.2, 7.1	18	15.3, 5.0
**I don’t eat fruits and vegetables because…**	**Frequency of “agree”**	**Weighted %, SE of %**	**Frequency of “strongly agree”**	**Weighted %, SE of %**
they cost too much	27	17.7, 4.7	10	16.9, 6.0
they often spoil before I eat them	40	36.0, 6.8	12	16.8, 5.6
they take too much time to prepare	9	7.0, 3.2	1	0.5, 0.5
they are not filling enough	19	22.5, 6.4	2	4.8, 3.4
my family doesn’t like them	16	14.5, 4.8	1	2.4, 2.4
the restaurants I go to don’t serve fruits	26	30.6, 6.8	3	5.8, 3.8
the restaurants I go to don’t serve vegetables	9	9.4, 4.0	0	0
I have trouble digesting them	9	4.3, 1.4	0	0
I don’t know how to choose fresh fruits and vegetables	9	12.7, 5.2	0	0
I don’t think of fruits and vegetables when looking for something to eat	16	15.4, 5.2	2	5.0, 3.5
they are too messy	4	2.1, 1.1	0	0

### Associations between farmers’ market use, and fruit and vegetable consumption and BMI

Among NC farmers’ market customers, in adjusted linear regression models with fruit and vegetable consumption as the dependent variable, there were no significant associations with farmers’ market use. Likewise, there was no association between BMI and either farmers’ market use and fruit and vegetable consumption.

Among KY farmers’ market customers, in adjusted models with fruit and vegetable consumption as the dependent variable, consumption was positively associated with use of farmers’ markets (estimate = 0.8, standard error = 0.4, p = 0.05). This means those who visited a farmers’ market at least 2–3 times a month consumed on average 0.8 serving of fruit and vegetable more than those who visited a farmers’ market at most once a month. There were no significant associations between farmers’ market use and fruit and vegetable consumption with BMI.

Among NC RDD respondents, in adjusted models with fruit and vegetable consumption as the dependent variable, consumption was positively associated with farmers’ market use (estimate = 1.3, standard error = 0.6, p = 0.03). Those who visited a farmers’ market at least 2–3 times a month consumed on average 1.3 servings of fruit and vegetable more than those who visited a farmers’ market at most once a month. There were no significant associations between the independent variables of interest and BMI.

Among KY RDD respondents, in adjusted models with fruit and vegetable consumption as the dependent variable, consumption was positively related to farmers’ market use (estimate = 1.0, standard error = 0.4, p = 0.02). Those who visited a farmers’ market at least 2–3 times a month consumed on average 1.0 servings of fruit and vegetable more than those who visited a farmers’ market at most once a month. There were no significant associations between BMI and the independent variables of interest.

Finally, the NC and KY RDD samples were pooled together and weights were adjusted by the states’ population sizes. In the adjusted model with fruit and vegetable consumption as the dependent variable, consumption was still positively associated with farmers’ market use within each state but the difference between the two states was not significant. There were no significant associations between BMI and the independent variables of interest, and there were no significant differences between the two states.

## Discussion

In this paper, not surprisingly, farmers’ market customers reported shopping more frequently at farmers’ markets compared to RDD respondents: Among farmers’ market customers, about half reported shopping at a farmers’ market at least once per week, compared to less than one fifth of NC and KY RDD respondents. This finding is in agreement with a previous study in Pitt County, finding that 17% of Pitt County, NC residents receiving food stamp benefits shopped at a farmers’ market, [15] and suggests that more work is needed to encourage county residents to shop at such locations. This is also similar to Racine, et al’s finding that 32.4% and 40% of Special Supplemental Nutrition Program for Women, Infants, and Children (WIC) participants in NC and Washington DC, respectively, had used a farmers’ market [29]. Furthermore, farmers’ market awareness scores were low, suggesting that efforts are needed to increase residents’ awareness of existing local farmers’ markets. Participants reported living a significant distance from farmers’ markets, which, coupled with lack of awareness, may present barriers to use of farmers’ markets.

Similar to our findings in eastern NC [16] and findings from studies in NC and DC [29] and in Florida among WIC participants [30], among the KY farmers’ market customers and NC and KY RDD respondents, fruit and vegetable consumption was positively associated with shopping at farmers’ markets. It is interesting to note, however, that farmers’ market customers reported consuming fewer fruits and vegetables, on average, than did RDD participants. This could be due to differences in survey administration (in person at the farmers’ market versus over the phone for the RDD survey). This difference could also indicate that farmers’ market customers are more health-aware in general, when compared to a representative sample of county residents, and thus may be better able to accurately estimate fruit and vegetable consumption. Counter to previous findings of inverse associations between access to farmers’ markets and obesity in an ecologic, national sample, [31] and in an individual analysis of eastern NC children from rural and urban areas, [32] we found no associations between farmers’ market use and BMI among farmers’ market customers or RDD respondents.

Our study findings should be interpreted with caution. This is a cross-sectional study design and thus demonstrates association and not causation. In addition, participant responses may have been influenced by social desirability bias, particularly among those sampled in-person at the farmers’ market, such that they overestimated healthy behaviors. However, farmers’ market customers may also have reported more accurately about healthy behaviors than RDD respondents. Farmers’ market customer recruitment methods may have led to systematic bias within the NC and KY farmers’ market customers. For example, farmers’ market customers who were willing to complete the survey may have been more likely to be female, higher socio-economic status, and thus able to spend more money at farmers’ markets, compared to those who were not willing to respond to our survey. In Pitt County, to increase survey administration efficiency, 25/70 customer surveys were completed by the customers versus by interviewers, and had incomplete responses, especially in terms of items in which an individual was supposed to mark only one choice. In addition, shopping patterns, fruit and vegetable consumption, and height and weight were self-reported among all respondents, and may be systematically biased. For instance, heavier individuals may under-report weight to a greater extent than normal weight individuals. Slightly different RDD methods were used in NC versus KY, but these methods were designed to be as consistent as possible, and the substantive benefits of conducting simultaneous analyses of the four samples in the two diverse rural areas outweighed the limitations. Another limitation is the small sample size, large standard errors, and lack of inclusion of potential confounders such as other dietary or physical activity factors that may influence BMI. Although we included cell phone numbers in the RDD survey, we may have had systematic bias in the sample. KY RDD response rate may have been higher than the NC RDD response rate because more call attempts were made in KY, and because the sample was older and only land lines were called. Finally, responses for the question regarding how often the respondents purchased fruits and vegetables locally grown from a farmers’ market, CSA (community supported agriculture), roadside stand, or pick-your-own produce farm may vary by the season in which the surveys were conducted, and may lead to an underestimation or an overestimation of the 12-month average.

Strengths of this study included use of the validated Block Fruit and Vegetable Screener, and RDD methods including cell phone numbers to select participants. Also, we assessed the frequency of farmers’ market shopping using typical behavior over the past 12 months, and since shopping at a farmers’ market may a fairly unusual experience for most people, such shopping may be easier to recall compared to other behaviors. Another strength of this study was the examination of rural populations, including two geographically diverse, rural southern populations, which have not been widely studied in farmers’ market research.

## Conclusions

The results provided here can assist in planning and evaluation of the NC Community Transformation Grant Project’s farmers’ market initiative, which has the goals of starting new farmers’ markets and making enhancements to farmers’ markets which include: creating or enhancing land use protections to support markets, improving physical structure of markets, increasing transportation to/from markets, and implementing SNAP EBT at markets. These enhancements are to be coupled with increased market promotion activities. Our results shed light on the farmers’ market enhancements that may be most needed in NC. First, to address the barrier of ‘out of the way’ location, more farmers’ markets are needed, including incorporating supports for farmers’ markets in land use planning and local zoning ordinances. Second, to address the barrier of ‘market days and hours’ , existing farmers’ markets should consider extending or rearranging hours to be more convenient to customers, and new markets should open during hours that existing markets are not open. As the two top scenarios that would encourage individuals to shop more frequently at farmers’ markets are more vendors and more promotional activities, both these enhancements should be explored. The knowledge gained from this evaluation can also be shared with other CTG-funded states, especially those states funded at the CTG-capacity building level, such as Kentucky, which were awarded funds to begin building infrastructure to apply for larger CTG implementation award.

There are many programs in place to facilitate SNAP EBT access at farmers’ markets, yet in our sample, less than 2% of all respondents reported that the lack of markets accepting SNAP EBT was a barrier to farmers’ market shopping. These results suggest that the cost-benefit ratio of implementing SNAP EBT access at all markets should be more fully examined before implementation in all markets, as this is a costly and time-consuming process. Fruit and vegetable consumption was positively associated with farmers’ market shopping among three of four rural samples. Thus, farmers’ markets may be a viable method to increase population-level produce consumption in rural areas.

## Competing interests

The authors declare that they have no competing interests.

## Authors’ contributions

SBJP and AG conceptualized and led data collection, analysis and manuscript writing. QW analyzed the data and contributed to manuscript writing. MLM and RKW collected data and contributed to manuscript conceptualization and writing. JTM contributed to manuscript conceptualization and writing. APR created sampling design and weights, and contributed to manuscript conceptualization and writing. MFL led the random digit dial survey methodology. KRE, TCK, and ASA contributed to manuscript conceptualization and reviewed multiple versions of the manuscript. All authors have read and approved the final version of the manuscript as submitted.
